# Supplementary material for: Tendinopathies and Pain Sensitisation: A Meta-Analysis with Meta-Regression
Source: Biomedicines. 2022 Jul 20;10(7):1749. doi: 10.3390/biomedicines10071749 (PMC9313266; doi:10.3390/biomedicines10071749)
Supplement: Supplementary file 1 [file biomedicines-10-01749-s001.zip › biomedicines-1786466-supplementary-done.pdf]

Table S1. Results of the risk of bias assessment with the Newcastle-Ottawa Scale.

Study	Adequate case definition	Representativeness of cases	Selection of controls	Definition of controls	Controlled for age	Controlled for other factors	Ascertainment of exposure	Same method for cases and controls	Non response rate
Alburquerque-Sendı´n, 2013	x	x	x	x	x	x	x	x	NA
Bisset, 2006	x	x	x	x	x	x	x	x	NA
Chimenti, 2020	x	x	x	x	x	x	x	x	NA
Coombs, 2012	x	x	x	x	x	x	x	x	NA
Eckenrode, 2018	x	x	x	x	x	x	x	x	NA
Fernàndez-Carnero, 2009	x	x	x	x	x	x	x	x	NA
Fernandez-Carnero, 2009	x	x	x	x	x	x	x	x	NA
Fernández-de-las-Peñas, 2010	x		x	x	x	x	x	x	NA
Ferrer-Pena, 2019	x	x	NA	NA	NA	NA	x	NA	NA
French, 2019	x	x	x	x	x	x	x	x	NA
Garnevall, 2013	x	x	x	x	x	x	x	x	NA
Gwilym, 2010	x	x	x	x	x	x	x	x	NA
Hamstra-Wright, 2020	x		x	x	x	x	x	x	NA
Hidalgo-Lozano, 2010	x	x	x	x	x	x	x	x	NA
Jespersen, 2013	x		x	x	x	x	x	x	NA
Karasugi, 2016	x	x	NA	NA	NA	NA	x	NA	NA
Ko, 2018	x	x	NA	NA	NA	NA	x	NA	NA
Kregel, 2013	x	x	x	x	x	x	x	x	NA

**Lagas, 2021**	x	x	NA	NA	NA	NA	x	NA	NA
**Lim, 2010**	x	x	x	x	x	x	x	x	NA
**Lim, 2017**	x	x	x	x	x	x	x	x	NA
**Paul, 2012**	x	x	x	x			x	x	NA
**Plaza-Manzano, 2019**	x	x	x	x	x	x	x	x	NA
**Plinsinga, 2017**	x	x	x	x	x	x	x	x	NA
**Plinsinga, 2020 (GTPS)**	x		x	x	x		x	x	NA
**Plinsinga, 2020 (PT)**	x	x	x	x	x		x	x	NA
**Riel, 2019**	x	x	x	x	x		x	x	NA
**Slater, 2004**	x	x	x	x	x	x	x	x	NA
**Tompra, 2016**	x	x	x	x	x	x	x	x	NA
**Ruiz-Ruiz, 2011**	x	x	x	x	x	x	x	x	NA
**Vallance, 2021**	x		x	x	x	x	x	x	NA
**Van Wilgen, 2013**	x		x	x	x	x	x	x	NA
**Wheeler, 2017**	x	x	NA	NA	NA	NA	x	NA	NA
**Wheeler, 2019**	x	x	NA	NA	NA	NA	x	NA	NA

NA: Not applicable.
